# Hospital Annual Meetings

**Published:** 1920-04-17

**Authors:** 


### Hospital Annual Meetings.

### Queen Charlotte's Hospital.

The total number of patients admitted in 1919 was 1,991, as compared with 1,649 a year previously—an increase of 342. On the out-patient districts 1,953 women were delivered in their own homes by the hospital midwives. Grants were received during the year from the Ministry of Health in aid of the District Midwifery and Ante-Natal Departments and the Infant Consultation Centre. Over 4,351 women attended the Ante-Natal Department during the period under review, as against 3,735 in 1918, while the number of children brought to the Centre rose from 171 in that year to 237 twelve months later. Expenditure last year increased by £2,694 to £15,820, while income amounted to £13,749, compared with £12,970 in 1918—an increase of £779, but insufficient to meet the outgoings by £2,071. The Committee has resolved to make an urgent appeal for sufficient funds to build a new out-patient department and effect improvements in the wards. With that object a special appeal committee has been formed for the purpose of raising £100,000, which it is estimated will be the cost of the extension and alterations necessary. To commemorate the visit of the Queen to the hospital last year her Majesty has presented the institution with signed portraits of herself and the King, which have been hung in the Board Room.

### National Hospital for Diseases of the Heart.

The number of out-patients' attendances during 1919 at the National Hospital for Diseases of the Heart, the annual meeting of which was held last week, under the presidency of Lord Verulam, constituted a record in the history of the institution, while new out-patients increased by over 900 to 3,126. Not less, it was estimated, than £10,000 would be required to provide the additional consulting rooms, dispensary, and equipment which are so urgently needed. The site for the out-patient extension has now been acquired and approved by the King's Fund, which has made a grant of £400 towards the cost mentioned. Income last year amounted to £6,943, as against £6,227 in 1918. Expenditure, however, increased from £6,242 in that period to £7,399 during the twelve months under review, and there was consequently a deficit of £456.

